# Sustainable food systems for optimal planetary health

**DOI:** 10.1093/trstmh/trx038

**Published:** 2017-10-16

**Authors:** Chelsey R Canavan, Ramadhani A Noor, Christopher D Golden, Calestous Juma, Wafaie Fawzi

**Affiliations:** a Harvard T.H. Chan School of Public Health, Boston, MA, 02115, USA; b Department of Global Health and Population; c Department of Nutrition; d Department of Environmental Health; e Department of Epidemiology; f Harvard University Center for the Environment, Cambridge, MA, 02138, USA; g Harvard Kennedy School, Cambridge, MA, 02138, USA; h Belfer Center for Science and International Affairs

**Keywords:** Agriculture, Environment, Food systems, Infectious disease, Nutrition, Planetary health

## Abstract

Sustainable food systems are an important component of a planetary health strategy to reduce the threat of infectious disease, minimize environmental footprint and promote nutrition. Human population trends and dietary transition have led to growing demand for food and increasing production and consumption of meat, amid declining availability of arable land and water. The intensification of livestock production has serious environmental and infectious disease impacts. Land clearing for agriculture alters ecosystems, increases human-wildlife interactions and leads to disease proliferation. Context-specific interventions should be evaluated towards optimizing nutrition resilience, minimizing environmental footprint and reducing animal and human disease risk.

Minimizing the negative environmental and health impacts of food system dynamics are central to a planetary health strategy to reduce the threat of infectious disease and promote healthy and sustainable diets and nutrition. A planetary health perspective recognizes that the health of human populations is tied to Earth's natural systems and biodiversity. Climate change influences the production, distribution and nutrient content of food.^1^ At the same time, agricultural production systems have important impacts on the environment and public health. Here we discuss agricultural production practices that impact planetary health and have implications for both the rise of non-communicable diseases and the spread of infectious diseases. We offer examples of solutions towards optimizing nutrition resilience (the ability to ensure adequate nutrition for global populations over the long term), minimizing environmental footprint (the impact of agricultural activites on natural resource use) and reducing animal and human disease risk.

Global population trends and dietary transition underlie the way food is produced. Unprecedented demand for diverse, safe and nutrient-rich food globally has become increasingly difficult to meet. While the past several decades have seen large increases in agricultural productivity, climate change and pressure on natural resources have hindered the ability of the agriculture sector to keep pace. This is especially true in developing countries with high rates of population growth and urbanization, which lead to changes in income levels and food preferences. Africa's urban population is projected to triple in the coming decades, with 1.3 billion people living in cities by 2050.^2^ A nutritional transition marked by increases in refined starches and other processed foods, meat, saturated and trans fats, and sugars accompanies urbanization and development, and is associated with rising rates of obesity and diet-related non-communicable diseases.^3,4^ Developing countries’ share of world meat consumption is expected to increase from 54% percent currently to 63% by 2050.^5^ They will increase their production of meat to 70% of the global total over this same period.^6^ Increased demand for meat and other foods is taking place amid declining availability of arable land and water scarcity.

These trends have led to the intensification of agricultural production, with serious environmental and infectious disease implications. Intensive livestock systems, where many animals are kept in close quarters, increases the risk for infectious diseases to proliferate and spread between animals. The response among producers in developed countries has largely been to increase the use of antibiotics in livestock. The overuse of antibiotics in this context is a key contributor to antimicrobial resistance, which poses an enormous threat to public health.^7^ Intensive livestock systems can also increase the risk of zoonotic disease emergence and transmission from animals to humans.^8^ In Malaysia in 1998, for example, the proximity of a pig farm to nearby fruiting trees allowed for animals to come into contact with bats carrying Nipah virus. An intensive production system, with over 30 000 animals, allowed for increased transmission between pigs, therefore increasing the likelihood of human infection with Nipah. Two hundred and sixty-five people were infected and over one million pigs slaughtered by the end of the outbreak.^9^ The origins of the global SARS outbreak of 2003 were traced to food animal markets in China. In addition, several zoonotic diseases, such as avian influenza, can infect poultry and spread quickly through large flocks. High rates of transmission increase the likelihood of a virus evolving with high pathogenicity and spreading to humans. Therefore, when a bird in an intensive production system becomes infected, entire flocks are culled, with implications for food security, livelihoods and animal welfare.

Growth and intensification of agriculture have also contributed to massive land-use changes. Livestock grazing uses 26% of arable land worldwide with another 33% used for growing crops for livestock feed.^10^ Land clearing for agriculture alters ecosystems, increases human-wildlife interactions and leads to disease proliferation. In Belize, for example, agriculture land-use and the overuse of fertilizers create favorable habitats for mosquitos that lead to increased risk of malaria.^11^ The emergence and re-emergence of several zoonotic and vector-borne diseases, including viral hemorrhagic fevers such as Lassa fever and Hantavirus, are linked to increases in human-animal interactions as a result of farming and deforestation.^12^ Land clearing also has implications for the natural environment, disrupting habitats and contributing to species extinctions. Importantly, livestock production, including animal lifecycle, land clearing for grazing and value chain for animal-source foods, is a massive contributor to worldwide greenhouse gas emissions. Climate change, in turn, impacts pollinators, plant diseases, water availability, soil erosion, rainfall and temperature, with serious consequences for agricultural production and food security.^13^

A planetary health approach to food systems should comprehensively address nutrition, infectious disease and environmental sustainability. There are several opportunities for interventions and policies to be adopted and rigorously evaluated in specific contexts. These may include incentives to reverse and reduce the upward trend in meat consumption in developed and developing countries and to promote dietary diversity for nutrition as well as biodiversity for conservation. For example, price controls on beef and nutrition education could lower demand for red meat in urban areas, reducing intake and risk for non-communicable diseases such as diabetes and certain cancers. At the same time, reducing food waste and creating innovative market structures, such as community supported agriculture or infrastructure for domestic and regional trade, will help reduce barriers to healthy foods and support livelihoods. Climate smart, nutrition-sensitive agriculture production strategies could also be employed to promote diversity and nutrient quality of crops, as well as production of highly nutritious indigenous varieties. Cadres of agricultural extension workers already exist in many developing countries and, together with community health workers, offer a promising avenue for improving production and consumption of diverse, healthy foods at the community level. Finally, safety standards and monitoring for intensive livestock production can help regulate antibiotic use, vaccination and waste management practices, as well as worker and animal welfare.

These strategies aim to reduce environmental degradation and the risk of infectious disease, non-communicable disease and persistent undernutrition. Unsustainable agricultural production is both a driver of climate change and a consequence of its effects. Intensification of livestock production and changing land-use practices exacerbate issues of poor nutrition and food insecurity, environmental degradation and the proliferation of infectious disease.^13^ In addition to nutritional impacts, sustainable diets have exponential benefits for the environment and public health. More research is needed to identify effective solutions and develop appropriate metrics for evaluation of food system interventions, especially in developing country contexts. Evidence-based and deliberate agriculture and public health programs are needed to ensure our food systems are promoting, not harming planetary health.
